# Breathing in an Accelerated World: Temporality, Ethical Repertoires, and Medical Students’ Examination Texts on Advanced Amyotrophic Lateral Sclerosis

**DOI:** 10.1007/s11013-026-10023-2

**Published:** 2026-11-13

**Authors:** Søren Bruno Elmgreen

**Affiliations:** 1 Department of Neurology, Aalborg University Hospital, Aalborg, Denmark; 2 Department of Clinical Medicine, Aalborg University, Aalborg, Denmark

**Keywords:** Amyotrophic lateral sclerosis, Medical education, Genre analysis, Temporality, Medical humanities

## Abstract

Medical students are commonly examined on ethically difficult clinical decisions, yet examination scripts have rarely been treated as a distinct empirical object in medical humanities. This article analyses 61 anonymised written examination responses from Danish medical students addressing acute respiratory failure in a fictional patient with advanced amyotrophic lateral sclerosis (ALS). The study is framed as a genre analysis of examination texts, operationalised through repeated close reading, inductive coding of recurrent textual moves, and abductive interpretation through Hartmut Rosa’s concepts of acceleration and resonance. Four ethical repertoires were identified: accelerated stabilisation; prognostic triage and the limit of intervention; distributed responsibility and the search for a legitimate pause; and fragile resonance through attention to wishes, dignity, and palliative alternatives. Across the corpus, students wrote judgement through temporal distinctions between urgency and delay, reversibility and progression, and escalation and proportionate limitation. The findings do not show professionalism as such, but rather the institutionally legitimate ways in which professional judgement is made legible under assessment. Examination scripts matter for medical humanities because they make curricular normativity and evaluative pressure textually visible, showing how future clinicians are taught to write responsibility, uncertainty, and care into the same ethical scene. These repertoires also render an ableist prognostic imagination institutionally legible—one in which ventilator-dependent life is readily scripted in advance as a future to be avoided rather than supported—linking the temporal analysis to critical disability studies’ account of the curative imaginary.

**Supplementary Information:**

The online version contains supplementary material available at https://doi.org/10.1007/s11013-026-10023-2.

## Introduction

Questions of time run through contemporary medicine. Acute care depends on speed, coordination, and the promise that technical intervention can avert deterioration before irreversible damage occurs. Yet clinical work is also filled with situations in which speed is not self-evidently good: the wrong tempo can overwhelm deliberation, curtail explanation, or transform treatment into the mere prolongation of biological process. Medical humanities scholarship has shown that temporality is not a neutral backdrop to care, but a constitutive dimension of illness, treatment, and moral life (Kaufman, 2015; Mattingly, 2014; Mol, 2008; Rosa, 2013, 2019).

Amyotrophic lateral sclerosis (ALS) brings these tensions into particularly sharp relief. Respiratory decline is common, and ventilatory support can relieve symptoms and, in selected circumstances, prolong survival; yet ALS remains progressive, and decisions about intubation or invasive ventilation often crystallise wider questions about reversibility, burden, quality of life, and the timing of death (Danel-Brunaud et al., 2017; Hobson & McDermott, 2016; Oliver, 2004; Phelps et al., 2017; Thorborg et al., 2023). What looks, in one moment, like life-saving escalation may look, in the next, like an intervention that buys time only by reorganising suffering. The issue is therefore not simply whether technology is available, but what kind of future that technology is imagined to open.

These tensions matter pedagogically as well as clinically. Medical students are trained to recognise deterioration early, escalate appropriately and act under pressure. At the same time, they are expected to engage prognosis, patient wishes and questions of proportionality. How these demands are articulated in training is an empirical question. Yet medical humanities research has devoted much more attention to patients’ time, waiting, chronicity, and dying than to the temporal norms through which future clinicians are socialised.

This article examines a corpus of 61 written examination responses from Danish medical students who were asked to address the ethical handling of acute respiratory failure in a fictional clinical vignette describing a 55-year-old woman with advanced ALS. The wider examination case moved from a focal peripheral neurological presentation towards a more diffuse pattern consistent with motor neurone disease, culminating in the question of respiratory failure and possible intensive care. The pedagogical format matters for medical humanities. Unlike interviews, reflective portfolios, or ordinary clinical records, examination scripts are written in order to be judged. They therefore condense curricular norms, genre expectation, and evaluative pressure into a tightly structured scene of professional legibility (Atkinson & Coffey, 2011; Bazerman & Prior, 2003). These texts are best read not as direct reports of stable attitudes, but as social texts in which students perform institutionally recognisable forms of ethical judgement.

The article develops two claims. First, temporality is not merely something students reason about; it structures the very form of their reasoning. Second, Rosa’s sociology helps clarify how the students’ responses are organised by competing temporal orientations: the imperative to accelerate, the recognition that acceleration reaches limits, and the intermittent attempt to reopen a more responsive relation to the patient. To avoid theoretical overclaim, I do not treat resonance as fully realised in the data. Rather, the scripts show fragile openings towards resonance—moments in which the student briefly suspends procedural momentum in order to ask what this intervention means for this patient, at this point in the illness trajectory.

The research question is therefore twofold: what ethical repertoires do students mobilise when a case of ALS culminates in possible intubation and intensive care, and what do those repertoires reveal about the temporal ordering of professional judgement as it is rendered legible under assessment?

### Hartmut Rosa, Temporality, and the Medical Profession

Rosa is not, of course, the only theorist of time relevant to this material. The temporality of medicine has been approached through the anthropology of clinical time and the ordinary work of prognosis and dying (Kaufman, 2015; Mattingly, 2014) and, more pointedly for the present case, through queer and crip theorisations of time, which foreground how disabled and chronically ill lives unsettle normative timelines of development, productivity, and cure (Jain, 2007; Kafer, 2013; Samuels, 2017; Wasson, 2021). These literatures are not interchangeable. Crip temporality theorises time from the standpoint of disabled life and its refusal of curative timelines; Rosa theorises the institutional time-regime into which students are being socialised. I foreground Rosa here for a specific reason: his account uniquely links macro-level institutional dynamics—dynamic stabilisation, and the way acceleration comes to function as professional legitimacy—to the micro-level orientations through which individual actors write and justify judgement. That linkage is what an institutional genre such as the examination script requires. The two framings are complementary rather than competing, and I return to their intersection in the Discussion.

Rosa’s work offers a useful vocabulary for analysing these texts because it links micro-level experience to institutional forms. In Social Acceleration, Rosa argues that modernity is marked by mutually reinforcing accelerations in technology, social change, and the pace of life (Rosa, 2013). The paradox of acceleration is that time-saving innovations do not usually create temporal abundance; instead, they raise expectations, intensify throughput, and produce chronic scarcity. In medicine, this paradox is familiar. Faster diagnostics, streamlined pathways, and expanded treatment options do not necessarily make clinicians feel less pressed. They often increase the number of things that can and therefore should be done.

For Rosa, this is not only a matter of speed, but of social reproduction. Modern institutions are sustained through what he elsewhere terms dynamic stabilisation: they must grow, innovate, and optimise simply to remain what they are (Rosa, 2013, 2019). Clinical practice is a paradigmatic site of such pressure. Professional competence is tied to responsiveness, escalation, and the capacity to keep pace with changing knowledge, protocols, and expectations. The medical profession is therefore not external to acceleration; it is one of the fields through which acceleration is enacted and normalised.

Rosa’s later concept of resonance names a qualitatively different mode of relation to the world (Rosa, 2019). Resonance is not slowness as such, nor is it sentimentality. It describes a relation in which a subject is affectively reached, responds, and is potentially transformed by the encounter. Resonance cannot be manufactured on demand and does not abolish technique or decision. Yet it is incompatible with treating the world as a fully available object of control. In clinical terms, resonance would not mean abandoning judgement, but recognising that good care may require receptivity to what is not exhausted by protocol: patient values, existential meaning, fear, ambivalence, and the irreducible uncertainty of outcomes.

This framework is attractive for medical humanities, but it needs careful use. Collapse is not a formal Rosaian category on the same level as acceleration and resonance. In the analysis that follows, I use collapse more modestly, as a shorthand for moments when the promise of accelerated intervention folds back on itself and appears as suspended dying, unwanted burden or a future stripped of meaningful restoration. This usage is grounded in the data, but it should not be mistaken for a strict triad in Rosa’s own system.

The framework also requires protection against two opposite errors. The first is to read every protocolled action as ethically impoverished. This would be untenable in acute medicine, where rapid stabilisation is often a precondition for any later conversation. The second is to romanticise every mention of wishes, dignity or family as evidence of resonance. In an examination script, such language may be formulaic, strategic, or curricular. The analytic task is therefore not to identify students who are “really resonant”, but to examine how the genre permits, constrains, or rewards certain ways of linking urgency, prognosis, and relational care.

Three features of Rosa’s thought are especially useful here. First, acceleration is an institutional and professional orientation, not just an individual feeling of being rushed. Second, acceleration is often experienced as morally legitimate, because to act quickly is to appear responsible. Third, resonance in professional settings is fragile and conditional; it often survives only where actors can create or defend a pause within an accelerative environment. These propositions are particularly apt for examination texts, where students must demonstrate decisiveness while simultaneously acknowledging the ethical remainder that decisiveness cannot eliminate.

## Methods

The material consisted of all 61 available written responses to the relevant question in a written examination in clinical neurology and neurosurgery for fifth-year medical students at Aalborg University. The examination presented a staged clinical case of a 55-year-old woman whose earlier neurological symptoms developed into a picture consistent with ALS and whose final deterioration involved acute respiratory failure; the response analysed here was its final sub-question (item 10 of 10), which addressed the ethical considerations facing a junior doctor in relation to intensive care, mechanical ventilation, and treatment level. The examination format matters for what these texts are. The neurological essay was one of two essays—neurological and neurosurgical, weighted 30% each—alongside ten short-answer questions (40% in total), and it was progressive: each sub-question was released only once the previous had been answered, so students reached the ethical question having themselves already worked the case from an undifferentiated hand weakness to a diagnosis of classic ALS, and having just stated its prognosis (a serious, progressive disorder with a mean survival of roughly three years from symptom onset). The ethical question was the only free-text, essay-style item calling explicitly for ethical reasoning; short-answer questions elsewhere required single words or brief factual description. Students were therefore clearly cued—by the demand for connected prose and by the wording of the prompt, which asked what “ethical considerations” a junior doctor faces and how “such a difficult situation” might be approached—that reflective ethical judgement, rather than factual recall, was being assessed. Unlike settings in which ethics is examined as a separate “ethics and society” component, ethical reasoning here was embedded within an integrated clinical case and assessed by clinician-examiners as one dimension of neurological competence.

This bears directly on the choice of genre analysis over, for instance, a discourse analysis of attitudes. Because students knowingly composed “the ethics answer” under cue and assessment, the analytic object is not what they privately believe about ALS but what the genre makes legible and rewardable as competent ethical judgement. The cueing is thus not a contamination of the data; it is part of the object of study. So too are the vignette and its marking guidance. The case arrived pre-labelled as classic ALS with an explicitly poor prognosis, and the examiners’ answer guidance foregrounded the “reversibility of the pneumonia and the risk of permanent ventilator dependence” alongside “clarification of the patient’s wishes regarding treatment level”. The institution, in other words, had already framed invasive ventilation as a risk of permanent dependence to be weighed rather than a mode of livable life to be supported—an observation to which I return in the Discussion.

The responses were short-form texts, typically between approximately 100 and 350 words, written under timed assessment conditions. This is methodologically important. This study is best understood as a genre analysis of examination texts. Following Atkinson and Coffey, I treat the scripts as documentary artefacts that do social work (Atkinson & Coffey, 2011). Following Bazerman and Prior, I approach them as genre-bound textual performances shaped by institutional expectation (Bazerman & Prior, 2003). The corpus therefore does not provide transparent access to what students privately think about ALS and intubation. Rather, it shows the institutionally legitimate ways in which ethical and professional judgement are written so as to become recognisable, credible, and assessable within this examination format.

The analysis proceeded in three steps. First, all 61 responses were read repeatedly in the original Danish and coded inductively, close to the data, for recurrent textual moves: references to acute action, treatment ceilings, prognostic reasoning, reversibility, responsibility, patient and family wishes, quality of life, dignity, palliative alternatives and descriptions of what life after ventilation might look like. Second, I compared these coded segments across the corpus in order to identify recurring combinations rather than isolated themes: patterned ways of framing the problem, assigning responsibility, imagining the future, and justifying action. It is these patterned combinations that I call ethical repertoires. Third, these repertoires were interpreted abductively through Rosa’s concepts of acceleration and resonance in order to specify the temporal logic organising them. This did not replace empirical coding with theory; rather, it clarified how the examination genre made certain relations between urgency, prognosis, pause, and patient-centred responsiveness textually available.

The analysis also paid attention to patterned frequencies, not as statistical outcomes but as an indication of which textual moves recurred often enough to constitute repertoires within the genre (Table 1). On this coding, 51 of the 61 responses referred explicitly to patient and/or family wishes; 40 referred to consultation with a senior colleague, specialist or back-up registrar; 19 explicitly imagined a prior conversation or documented treatment ceiling; nine raised potentially reversible contributors such as pneumonia; and 13 mentioned palliative alternatives such as morphine, comfort care, or a dignified death. These categories overlap and are not exhaustive, but they help show which combinations of concern, responsibility, and temporal orientation were institutionally recognisable across the corpus.

**Table 1:** Recurrent motifs across the 61 examination responses

Motif	*n*	Typical formulation	Analytic significance
Patient and/or family wishes	51	“What does the patient want?”; “talk with patient and relatives”	Resonance-oriented opening; relational accountability
Senior colleague/specialist consultation	40	“I would call the back-up registrar/senior doctor”	Distributed responsibility under temporal pressure
Prior discussion or documented treatment ceiling	19	“This should have been discussed beforehand”; “check the chart”	Moves ethical labour upstream in time
Potentially reversible contributor	9	“Could this be pneumonia?”	Complicates simple futility judgements
Palliative alternative explicitly named	13	Morphine, comfort care, “good/decent death”	Limit-experience of intervention; opening towards non-escalatory care

Quotations were translated from Danish into English by the author for the purposes of publication. The translations aim to preserve meaning and tone rather than produce literary fluency. Minor grammatical irregularities in the original have been lightly smoothed where necessary for readability. Student identifiers are reported only as numeric codes.

The material was fully anonymised before analysis. No patient-identifiable information was present in the dataset, and the student responses were analysed as secondary educational texts rather than as material generated prospectively for research. Even so, the interpretive extension from assessment to scholarship raises ethical questions about context and representation. For that reason, the analysis proceeds at the level of collective repertoires rather than individual competence, and quotations are used sparingly and only insofar as they illuminate recurrent patterns.

## Results

The corpus did not divide neatly into pro-intubation and anti-intubation positions. Instead, the responses showed a recurrent oscillation between urgent action, prognostic restraint, and relational concern. Four repertoires were particularly prominent.

### Accelerated Stabilisation

The first repertoire was the reflex of escalation. Many students began from the practical demands of acute deterioration: airway and breathing assessment, oxygenation, non-invasive support, contacting intensive care, checking whether resuscitation status had already been documented, and seeking immediate senior input. Even when the responses moved quickly towards prognostic reflection, they often opened in the tempo of emergency medicine.

This repertoire was not reducible to crude technicality. It frequently represented the student’s attempt to appear responsible in an acute situation. One student wrote that this was “a life-threatening situation” in which one should quickly evaluate treatment level and involve more experienced colleagues. Another emphasised that if CPAP could stabilise the blood gases and oxygenation, “we have time to make a well-considered decision” (student #219823). In other words, temporally accelerated action sometimes functioned as a way of creating deliberative space. Intervention and reflection were not simply opposites.

At the same time, the genre rewarded concise procedural competence. When students described what the junior doctor should do, they often moved rapidly to the hierarchy of escalation: call the back-up registrar, involve anaesthesiology, contact intensive care, optimise while awaiting help. Forty of the 61 responses explicitly invoked such senior consultation. This recurrent pattern suggests that the scripts staged professionalism as distributed and vertical: good judgement involved recognising one’s limits and placing the case within the right chain of command.

### Prognostic Triage and the Limit of Intervention

If accelerated stabilisation defined the opening move, the dominant substantive concern was prognosis. Students repeatedly asked not only whether ventilation could be initiated, but what kind of future it would produce. Many imagined the respirator not as a bridge back to ordinary life, but as an intervention that the patient might “never come off … again” (student #223894). Another student wrote that intensive care was “for people with an expected reversible cause”, which ALS in itself was not (student #225851). Such formulations condensed a complex ethical judgement into a temporal one: does this intervention lead back to recoverable life, or only forward into prolonged dependency?

This was where the possibility of collapse emerged most strongly. In these scripts, the ventilator often appeared as a machine that can keep time going without restoring agency, function, or communicative life. Students worried about muscular exhaustion, extended rehabilitation, permanent dependence, and the possibility that treatment would return the patient to “a worse existence” during a short remaining lifespan (student #239070). One asked: “In what state would the patient wake up again? Is it dignified?” (student #242444). Another wrote bluntly that respirator treatment might merely “prolong the unavoidable fatal outcome” (student #222558).

Yet the students were not uniformly pessimistic. A minority retained an interventionist stance, especially when they foregrounded the patient’s age or the possibility of a reversible superadded illness. Nine responses explicitly raised pneumonia, infection, or another reversible contributor to the acute deterioration. One response argued that if the respiratory crisis was due to pneumonia, ventilation might be justified until the infection was treated (student #229675). Another described the case as a “borderline situation” in which, absent a prior decision about treatment level, the student would “let doubt benefit her and treat intensively” (student #233670). These responses matter because they show that the moral difficulty was not only futility, but uncertainty. Students were not merely sorting cases into treatable versus untreatable; they were trying to decide whether the present crisis belonged to the ordinary reversibility of acute medicine or the irreversible temporality of late-stage ALS.

### Distributed Responsibility and the Search for a Legitimate Pause

A third repertoire concerned authority, timing, and moral burden. The students repeatedly displaced final responsibility upwards or outwards—to a senior neurologist, an anaesthetist, an intensive care doctor, or a prior documented treatment decision. This was not simply defensive practice. It was also a way of seeking a legitimate pause in a compressed situation.

Student #223894 wrote: “I would always confer with a more experienced colleague, because at my stage of training I would not feel able to decide this on my own.” Similar responses did more than signal inexperience. They showed how ethical reasoning is organised institutionally. The “right” answer to moral difficulty was often not to decide alone, but to suspend solitary agency and bring the question into a recognised collective forum. Forty responses explicitly mentioned a senior colleague, and 19 imagined that this crisis should ideally have been preceded by an earlier discussion or documented treatment ceiling.

This temporal displacement is analytically important. Many responses suggested that the truly ethical act was not only to make the right decision in the acute moment, but to have moved the conversation earlier in the illness trajectory. Students wrote that discussion of ventilation, intensive care, and resuscitation should occur “beforehand”, “early in the course” or at the time of diagnosis. The emergency thus exposed an earlier temporal failure: the crisis becomes morally unmanageable when anticipatory planning has not happened. In Rosaian terms, the scripts repeatedly tried to move ethics upstream, away from the compressed tempo of emergency and into the slower time of planned conversation.

Seen from a slightly different angle, this repertoire is not only about displacing responsibility upward; it is also about receptivity. To call the senior registrar, to bring the case into a recognised collective forum, is to allow one’s own judgement to be reached and potentially altered by a perspective from outside the compressed situation—an orientation that research on clinical supervision and help-seeking has long treated as a competence rather than a failing (Kennedy et al., 2009). In Rosa’s terms this is proto-resonant: resonance is precisely the capacity to be addressed and moved by something not fully under one’s own control. The search for a legitimate pause is, in part, the search for a relation in which the case can speak back.

### Fragile Resonance: Wishes, Dignity and Palliative Alternatives

The most interesting passages in the corpus were those in which students briefly broke with a purely escalatory or prognostic register and asked what the intervention would mean for this patient’s lived world. These moments should not be over-romanticised. In examination writing, the language of wishes, dignity and empathy can be highly conventional. Even so, they represent openings towards a different temporal and moral orientation.

Fifty-one responses referred explicitly to patient and/or family wishes. In some cases, this language remained formulaic: the doctor should ask what the patient wants, inform the family, and weigh their views alongside medical judgement. In others it became more existential. Student #211670 wrote that the doctor should discuss “how the patient would prefer to spend her last time” and preserve her dignity “as much as possible”. Student #222558 asked directly: “What is a dignified death? And what is a good death?” Student #217169 emphasised not only explanation but empathy and respectful conversation with the patient or spouse.

Thirteen responses explicitly mentioned palliative alternatives—morphine, comfort measures, palliation or what several students called “kærlig pleje”, literally loving care. This language signalled recognition that not acting invasively may itself be an active mode of care. Student #219872 wrote: “Patients should not be kept alive just because we can, if there is no prospect of improvement and treatment only drags things out and does more harm than good.” In such passages, the imagined good doctor is no longer simply the one who escalates fastest, but the one who can bear the moral weight of non-escalation and still remain responsive.

Across the corpus, then, resonance appeared less as a stable ethic than as a fragile practice of reopening the case to dialogue: ask what was discussed earlier, ask what future the patient regards as acceptable, ask whether symptom relief rather than prolongation is now the more faithful form of care. These moments did not abolish acceleration; they interrupted it, however briefly.

## Discussion

The central finding is that the students’ responses are temporally stratified. They are not merely statements about ethics in ALS; they are institutionally shaped ways of writing judgement under assessment. Across the corpus, students repeatedly organised the case by distinguishing between urgent action and delayed conversation, reversible episode and irreversible progression, decisive escalation and ethically necessary pause.

This is where Rosa’s framework is most useful. The scripts show acceleration not as a pathology external to medicine, but as the ordinary moral infrastructure of competent clinical action. To recognise deterioration, to call intensive care, to optimise quickly, to move up the chain of command—these are not morally thin behaviours. They are precisely what the profession teaches. The students’ writing demonstrates how deeply this orientation has already been institutionalised by the time of late undergraduate training.

At the same time, the corpus shows that students do not simply inhabit an accelerative professional self without remainder. Many explicitly asked whether intensive care would return the patient to an acceptable life, whether a reversible cause was present, whether treatment would merely prolong suffering, and whether the conversation should have happened earlier. The most striking feature of the corpus is therefore not pure acceleration but oscillation. Students move between the demand to intervene, the recognition of limit, and the wish to reopen dialogue. This oscillation is a more convincing empirical finding than any rigid categorisation of students into separate moral types.

This oscillation can also be described in hermeneutic terms. The students move between the known—the protocol, the reversible episode that acute medicine is built to treat—and the unfamiliar—the irreversible temporality of dying with advanced ALS, which the protocol cannot resolve. Resonance, in Rosa’s sense, is itself rooted in this hermeneutic tradition: it names the moment at which the unfamiliar is allowed to address the writer rather than being assimilated to the known (Svenaeus, 2000). A fuller hermeneutic reframing lies beyond this paper’s single-framework design, but the oscillation the corpus displays is, at bottom, this fluctuation between familiarity and its limit.

The data also refine how resonance should be used in this context. A weaker manuscript would claim that some students achieved resonance. The present analysis does not need that claim. Examination texts cannot show whether students are in a resonant relation to patients in Rosa’s fuller sociological sense. What they can show is that the genre still contains resonance-oriented vocabulary and reasoning: patient wishes, a good death, dignity, the need to create time for a considered decision, the importance of prior conversations, the possibility that comfort rather than escalation may be the more responsive form of care. In a high-pressure examination, even these brief openings are significant because they indicate that relationality has not disappeared from the repertoire of legitimate medical judgement.

Another important finding concerns uncertainty. Much of the corpus rejected invasive ventilation on prognostic grounds, but a smaller set of responses foregrounded reversibility, especially infection or pneumonia. This matters because it shows that the ethical problem was not reducible to a binary between “treat” and “let die”. Students were reasoning within the ambiguity of acute-on-chronic illness, where the same intervention can be imagined as either temporising bridge or burdensome prolongation depending on how the present episode is temporally framed. The students’ recurrent question—what life are we returning her to?—is thus not only a question about values; it is also a question about clinical temporality and prognostic imagination.

### Prognostic Imagination and the Curative Imaginary

This prognostic imagination deserves closer scrutiny, because it is not temporally neutral. Several of the formulations quoted above—that ventilation would return the patient to “a worse existence,” the question “In what state would the patient wake up again? Is it dignified?”, the worry that treatment would merely “prolong the unavoidable fatal outcome”—trade on a cultural script in which ventilated, tracheostomy-dependent life is imagined in advance as a life not worth living. Critical disability studies gives this structure a name. What Clare (2017) calls the ideology of cure, and what Kafer (2013) analyses as the foreclosure of imagined disability futures, describe a horizon in which the only intelligible good outcome is restoration to independent, productive embodiment, and in which continued life with profound disability figures as mere prolongation. Jain’s (2007) account of “living in prognosis” and crip theorisations of non-normative time (Samuels, 2017; Wasson, 2021) show how deeply such futures are pre-scripted by medical temporality itself.

Empirically, this imagined future is at least contestable. Studies of people living with ALS on tracheostomy ventilation report self-assessed quality of life considerably higher than clinicians and families tend to predict, even where the burden on caregivers is substantial (Kaub-Wittemer et al., 2003), a divergence echoed in qualitative work on invasive home-ventilation decisions (Thorborg et al., 2023). The point is not that invasive ventilation is the right choice in this vignette—the case is deliberately built around genuine prognostic uncertainty—but that the students’ pauses, for all their ethical seriousness, frequently reopen the case towards a good death rather than towards the possibility of a livable ventilated life. The remainder that acceleration cannot absorb is repeatedly resolved in one direction; livability with severe disability rarely enters the frame as an imaginable future at all.

This reading does not displace the temporal analysis so much as sharpen it. If examination scripts make institutional normativity textually legible, then an ableist prognostic imagination is part of what this genre renders legible and rewardable as competent judgement—reinforced, as the marking guidance shows, by an assessment frame that itself names permanent ventilator dependence as a risk. Acceleration and the curative imaginary are, in this sense, two faces of the same institutional temporality: intervention counts as meaningful when it can be imagined to lead back to recoverable, independent life, and falters when it cannot. The methodological guardrail applied to resonance holds here too. These texts license claims about which imaginaries are institutionally legible, not about whether individual students hold ableist attitudes; the scripts reproduce a curative imaginary as assessable competence, which is not the same as showing that their authors are ableist.

The prominence of distributed responsibility is likewise revealing. One could read frequent reference to senior colleagues as mere insecurity. That would be too simple. These responses show that moral labour in medicine is organisationally mediated. Students have already learned that grave decisions about treatment limitation must be collectivised, documented, and legitimated through recognised hierarchies. The repeated wish that the issue had been discussed earlier suggests that what students find most ethically precarious is not only the content of the decision, but the timing of it. Ethical reflection becomes compressed and brittle when it is deferred until physiological crisis.

For medical education, this has at least three implications. First, anticipatory conversations about treatment level should not be taught as an optional communication add-on, but as a core temporal skill: a way of moving ethically difficult decisions into a time frame where dialogue is possible. Second, assessment should test not only whether students know when to escalate, but whether they can articulate the difference between reversible crisis and progression within terminal disease, including the uncertainty that often exists between those poles. Third, curricula should legitimate palliative and relational reasoning as professional competence rather than as the soft supplement to technical medicine. They should also make room for disabled and ventilator-using people’s own accounts of livable life, so that judgements about “quality of life” are formed from more than the curative imaginary and its imagined futures (Clare, 2017; Kafer, 2013). In the scripts, resonance became most visible when students created a pause—through CPAP, consultation, chart review or conversation—in which the patient’s world could become relevant again.

The article also has limitations. The material comes from a single institution and a single examination genre. The responses were written under assessment conditions and do not license strong claims about what these students would say or do in real clinical encounters, still less about professionalism as such. Nor can the present analysis determine how particular formulations were rewarded in marking. The purpose is narrower: to analyse the institutionally legitimate ways of writing professional judgement in this specific pedagogical setting. A further limitation follows from the choice of framework itself: reading the corpus through Rosa’s institutional lens foregrounds temporality and necessarily leaves other patterns in shadow. A sustained analysis through critical disability studies—one that systematically recoded the corpus for the workings of ableism and the curative imaginary—would foreground different features and constitutes a distinct study, which these anonymised examination texts would genuinely support; the present paper indicates that intersection without exhausting it.

These limitations do not undermine the specific claim made here; in some respects they clarify it. Precisely because the texts are stylised, they make institutional normativity textually visible. Examination scripts are important empirical objects for medical humanities not despite but because of their artificiality: they condense curriculum, genre expectation, and evaluative pressure into a form in which medicine stages and assesses recognisable professional judgement. They therefore provide a distinctive window onto professional formation—not access to authentic moral interiors, but insight into how responsibility, proportion, temporal reasoning, and patient-centred responsiveness are made legible before students become fully credentialled clinicians.

## Conclusion

The examination responses analysed here show that ethical reasoning about intensive care in advanced ALS is inseparable from questions of time. Across the corpus, students repeatedly made the case intelligible through temporal distinctions: urgent stabilisation versus delayed conversation, reversible episode versus irreversible progression, and symptom relief versus burdensome prolongation. The scripts therefore reveal recurring ethical repertoires through which judgement is written in this assessment setting.

Read through Rosa, these texts illuminate not medical professionalism as such but the temporal ordering of professional judgement as it is rendered legible under evaluation. Acceleration appears as a default moral competence: to act quickly is to appear responsible. Collapse appears when intervention can no longer be imagined as restoring a meaningful future. Resonance appears only intermittently and provisionally, as attempts to reopen the case to wishes, dignity, dialogue, and proportionate palliation. Those attempts, however, largely unfold within a curative imaginary that struggles to picture ventilated, disabled life as livable, so that the pause, when it comes, tends to open towards a good death rather than a supported life. Naming that horizon—and the institutional temporality that sustains it—is part of what makes examination scripts worth reading.

For medical humanities, the article suggests that examinations are not merely pedagogical instruments but valuable empirical sites for studying how institutions script and assess legitimate forms of judgement. Examination texts show how future doctors learn not only what to know, but how to textualise responsibility, temporal reasoning, and limits of intervention in ways that become recognisable as prudent and professional under evaluative pressure.

## Supplementary Information

Below is the link to the electronic supplementary material.

Supplementary file1 (DOCX 97 kb)

## Data Availability

The anonymised English translations of the examination responses analysed in this study are available in the Supplementary Information accompanying this article.
