# Supplementary material for: Breathing in an Accelerated World: Temporality, Ethical Repertoires, and Medical Students’ Examination Texts on Advanced Amyotrophic Lateral Sclerosis
Source: Cult Med Psychiatry. 2026 Nov 13;51(1):6. doi: 10.1007/s11013-026-10023-2 (PMC13768226; doi:10.1007/s11013-026-10023-2)
Supplement: Supplementary file 1 [file 11013_2026_10023_MOESM1_ESM.docx]

English Translation of Student Responses

Note. This document presents only the English translation of student responses. Respondent ID numbers have been preserved. Abbreviations have where possible been translated into plain English (for example, “BV” as “senior on-call doctor”), while clinical abbreviations such as NIV and CPAP have been retained.

| **Respondent ID** | **English translation** |
| --- | --- |
| 233304 | This is a difficult situation, in which consideration must be given to how the patient should best be treated, whether any treatment would benefit the patient, and at the same time how the relatives should be managed and informed so that they feel included throughout the process and that their views are also taken into account in the doctor’s deliberations. The prognosis of the disease is poor, and being placed on a ventilator is also very demanding, as rehabilitation after ventilation is prolonged and requires a certain degree of strength and health. Therefore, this should be discussed in collaboration with the patient, the relatives and more senior colleagues, in order to offer the best and most beneficial treatment. |
| 242444 | Has there been a conversation about resuscitation and ventilator treatment/intensive care? Does the patient have a wish? In what condition would the patient wake up again? Is that dignified? What sequelae will the patient develop, and how great a burden will ventilator treatment be? Can the patient come off the ventilator again at all? What rehabilitation potential is there? Discussion with colleagues/the senior on-call doctor with more experience to obtain advice and additional perspectives is important. Knowledge of the prognosis and the significance/consequences of the interventions is important. |
| 223894 | In this situation I would always discuss the matter with a more experienced colleague, because at this point in my training I would not feel able to decide this on my own. This patient group generally ends up requiring ventilator treatment, but that also entails considerable care needs. One must always weigh up what one believes to be the best and most reasonable outcome for the patient. Is a life on a ventilator something the patient wants? Once she is started on ventilator treatment, she will probably never come off it again. It may therefore be a good idea to have this discussion with the patient and any relatives early in the course. |
| 241784 | The assessment concerns whether the patient is a candidate for intensive care and resuscitation. It is extremely hard on the body, and the longer the stay in intensive care, the longer the rehabilitation required. Is there any prospect of improvement if she is admitted to intensive care? If the patient improves during the intensive care admission, one must consider what sort of life she will return to. Will the symptoms be worse than before? Is it a dignified life? Is it what the patient herself wants? As a junior doctor, I would discuss this with a consultant. These considerations can be discussed with the patient and the relatives. |
| 219823 | She is relatively young but has only a short life expectancy left. If she is placed on a ventilator, she will probably not come off it again, because she is already very weak and has no possibility of rebuilding her strength. She is muscularly exhausted, and a ventilator could relieve that. Normally this would give other people time to recover, but here it is not certain that it would. I hope that a decision had already been made before this situation arose as to whether she should be a candidate for ventilation and resuscitation. To wash out some of the CO2 we can give CPAP and immediately contact the senior on-call doctor and the relatives. CPAP may help her acid–base status, and if she can maintain her oxygen saturation, we have time to make a considered decision. In such a situation I would share my reflections with the nursing staff, the senior on-call doctor and, of course, the relatives. |
| 234154 | I would look in the patient’s notes to see whether she had expressed a view on resuscitation. In addition, I would speak with the relatives and ask what they had discussed with the patient if such a situation were to arise, and what their thoughts are about it. The patient’s future prospects of ending up in a wheelchair attached to a ventilator must also be taken into account and weighed against what kind of life it would be good for the patient to return to. |
| 235420 | 1) Have a conversation with the patient or the relatives about what they want. 2) In the end it is the anaesthetists who assess whether the patient is a candidate for intensive care, so they should of course be contacted. Ethical considerations include the fact that it is a major intervention for a patient to be admitted to intensive care and possibly placed on a ventilator, and the patient may never come off it again (here we do not know whether she has been re-admitted because of, for example, pneumonia, which might be treatable and thereby reduce the respiratory workload); on the other hand, several people with ALS in later stages do live with a ventilator, so it could be a permanent solution for her. |
| 196134 | I would consider whether or not the patient is a candidate for resuscitation and treatment in intensive care. This is a medical decision, but it is important to speak with the patient and the relatives about it, and in such a situation I would probably discuss it with my senior on-call doctor. The patient needs ventilatory support so that she can eliminate CO2. |
| 207311 | This should be done with a calm mind. One should check with the patient in advance whether they wish to be intubated or resuscitated, and have that information ready. After that, it must be done as professionally as possible, and I must do what is required of me. If I am in any doubt at all, I would first contact the senior on-call doctor regarding the ethical aspects. |
| 242232 | The patient has a right to treatment, but one must always consider whether one is doing more harm than good, and what the course after intensive care treatment (intubation/coma) would involve. As a very junior doctor, I would discuss this with a colleague. This is also something that could in fact have been discussed earlier with the patient if, for example, they had particular wishes. |
| 233631 | Here it is important to have spoken with the patient beforehand regarding wishes about intensive care, resuscitation and ventilator use. Continuous oxygen support may be possible afterwards. However, one must bear in mind that this is a progressive condition that will get worse and worse. Therefore, it is important to think about quality of life and the patient’s wishes. One must also assess whether, given any comorbidities, there is an indication for intervention and whether she would be able to recover afterwards. |
| 235879 | This is part of medical life. One should not expect all patients to come through well. The best one can do is to give them the treatment that is available and inform both the patient and the relatives about the course, even if it goes in the wrong direction. In such a difficult situation it is important to show care and understanding towards the patient and the relatives. One can also turn to older, more experienced colleagues and ask for good advice. |
| 233311 | In such a situation it is important to have clarified beforehand whether the patient wants ventilation and resuscitation. This should be done in collaboration with the patient, but in the end it is a medical judgement based on various factors such as prognosis and current functional status. This is a difficult ethical situation, because the patient may be so unwell in her usual functional state that one has had to decide on no intensive care and no resuscitation. One should try to help the patient as far as possible, and if the patient has not been placed on a palliative pathway, one should call anaesthetics/intensive care and otherwise optimise according to the ABCDE approach in the meantime. |
| 238069 | The patient does not have many years left to live, and depending on how the patient and her family feel, it may be ethically wrong to prolong her life further if that means she ends up only being able to lie attached to a ventilator while fully conscious. On the other hand, it may also be wrong to do nothing in a situation where her respiratory problem might possibly be improved. It might be a good idea to ask the patient and the relatives about this. In addition, as a junior doctor one should probably contact one’s senior on-call doctor or discuss the matter with an older colleague, possibly also colleagues from intensive care, in order to help the patient as well as possible. The patient and the relatives should certainly be informed of the possibility that she may never come off ventilator treatment again. |
| 235481 | Ethical considerations may include the indication for resuscitation, given that the patient has high morbidity with an ALS diagnosis and that this may be unjustifiable for the patient’s subsequent wellbeing. A prior conversation with the patient and relatives is important. Such a difficult situation can be discussed with more experienced senior on-call colleagues. There is also the psychological stress/difficulty of the situation, with possible psychological support for the doctor. |
| 234041 | The wishes of the patient and the relatives regarding intensive treatment are important. The benefit of ventilation is questionable: the patient will never recover from the need for treatment; ventilator treatment prolongs life but does not improve the prognosis—i.e. it buys time for the family and the patient. Accumulation of CO2 leads to CO2 narcosis, during which the patient can ‘quietly sleep’ into death. |
| 239070 | One must consider functional status, expected remaining life span and comorbidities in order to ‘calculate’ a prognosis on the other side of intensive care/ventilation, assuming the patient survives it at all. ALS is a disabling diagnosis in which the patient can never expect normal function, whether admitted to intensive care or not. After intensive care the patient may be even more disabled, and one may thus have caused a worse existence during her short remaining life. Her current day-to-day functional level is not known, and she is also only 55 years old. Depending on her functional level, the case could argue for or against intensive care, but her age is a factor in favour. The senior on-call doctor should be contacted regarding the treatment level. |
| 225851 | Intensive care is for people who have an acute illness with an expected reversible cause, which ALS does not provide. My thoughts are that, on the one hand, I want to do everything I can so that the family can reach her and say goodbye, but at the same time I do not want to subject a patient to unnecessary life-prolonging treatment if the quality of life that comes out of it is prolonged intubation in a bed without any prospect of a reversible cause and improvement. I do not know whether one would do this in a situation that has developed so quickly, in order to allow the relatives time to come and say goodbye, but I doubt it. My initial view would therefore be that the patient is not a candidate for intensive care. I would, however, always ask an older colleague in such a case, where I have an assumption but am not completely certain. |
| 211670 | I am thinking that the patient would not be a candidate for either intensive care or resuscitation, because of the rapid progression and the poor prognosis of the disease. One also has to think about what quality of life and capacity the patient still has. I would think about what would be most appropriate for the patient and what way through this situation would be best for her. I would involve the patient and possibly the relatives in the decision about how, and how aggressively, we should treat the patient. Here I would address how the patient would prefer things to proceed from here and how she would prefer to spend her final time. I would talk about making it as comfortable as possible for the patient and preserving her dignity as far as possible. I would explain what a stay in intensive care could entail, and what kind of life she could look forward to afterwards. |
| 221004 | Because ALS is a progressive disease, the patient’s respiratory muscles will eventually be affected (in addition to all the other muscles), and the patient will then need a ventilator because she cannot breathe on her own. This is unavoidable, and perhaps she has reached that stage now, but that is not entirely clear. It is also possible that her pneumonia is causing the deterioration and that we can treat that. Therefore, it is also uncertain whether she could come off the ventilator again afterwards and still have an acceptable life for some months to years, albeit with reduced quality of life, since the disease is progressive and will affect many aspects of her life—among other things she will eventually become immobile. However, there is a risk that she will not be able to come off the ventilator. Therefore she and her spouse must decide whether she should go on a ventilator and thereby have a chance of life-prolonging treatment, despite the substantial risk that she will never come off it again, or whether she should be allowed to drift peacefully away. This is a very difficult ethical dilemma, because as doctors we ought to do everything we can for her survival and therefore ought to ventilate her. But it may not be in her interest to live the rest of her life on a ventilator. This is a conversation we need to have with them, and they need to have with each other. It is her decision; we cannot make it for her. In addition, I need help from my senior on-call doctor. Perhaps the patient and spouse may also need to speak with a chaplain. But it is a time-critical situation, so that may not be so easy. |
| 234225 | Because we know that ALS has a poor prognosis, one would hopefully already have spoken with the patient and relatives about the disease, its prognosis, and wishes concerning ventilator treatment and/or resuscitation before a situation like this arises. My considerations would concern the individual prognosis, the patient’s wishes, what kind of life the patient can expect, and what is ethically defensible as a doctor. I am a junior and inexperienced doctor, so I would urgently discuss the matter with the neurology senior on-call doctor and anaesthetics and tell them my reflections. Presumably the patient has also been discussed at a multidisciplinary conference where possible outcomes and treatment options were considered. |
| 220587 | There is no prospect that the patient will come off the ventilator well again. Therefore, one can consider whether the burden of anaesthesia, if required, is the right thing for the patient and the relatives. A conversation should be held with the relatives—and with the patient if she is able to take part. |
| 220427 | It is important that, as far as possible, in ‘peacetime’ one has already spoken with the patient about ventilation and resuscitation in the event of acute illness and deterioration. Because the patient has ALS, it is unlikely that she will become ‘strong’ enough to come off ventilator treatment once it has begun. When using a ventilator it is important to assess the patient’s level of functioning before the disease progressed, and whether it is realistic that the same level of function could be regained after the illness. There is a prolonged rehabilitation course after ventilator treatment, which may be difficult for a patient who is already weakened to complete. Unfortunately, with ALS there is a high risk that the patient will lose many functions during ventilator treatment. At the same time one must consider the patient’s age and family situation. Are there many children at home? Does the patient wish to be present for a particular event (a summer wedding, confirmation, graduation celebration, christening, etc.)? Is it possible to accommodate such wishes through a short admission to intensive care? And is the patient well informed about the risks associated with ventilator treatment? |
| 221332 | This is a life-threatening situation in which the ABCDE approach should be used to assess severity. A decision must be made as to whether it is defensible for the patient to receive intensive care treatment, and also whether she is a candidate for attempted resuscitation and for ventilation (it requires extensive rehabilitation to come off a ventilator). One can therefore assess the situation by evaluating the patient’s Clinical Frailty Scale score, comorbidities, and the progression/stage of the classical ALS. As a junior doctor, I would of course also contact the senior on-call doctor. Ultimately it is a medical judgement whether the patient is a candidate for the various intensive treatments, but one can also speak with the patient about the seriousness of the situation and her own wishes/expectations, because it may be that she does not in fact wish to continue if the outlook is severe deterioration and no quality of life, in which case it is better to contact the relatives so that life can end with dignity among those closest to the patient, along with palliative treatment. |
| 239996 | I would start with an ABCDE assessment. When I do not know what should happen or what is right, one must call for help and reach out. I would contact my colleagues, and I would contact my senior on-call doctor and follow their advice. It is also important to remember to say when one needs that help, so that mistakes do not occur. It is my decision even if the nursing staff are pressing, and here one has to try to keep a cool head, but I would ask the senior on-call doctor to come. |
| 242547 | It must be assessed whether the patient is a candidate for intensive care and/or resuscitation, because the patient is severely affected and would have to be intubated and ventilated if she is to survive. Given the patient’s relatively young age, one can argue that she is a candidate for both intensive care and ventilation. Depending on the severity of her ALS, however, one could also argue that she would not be. If one judges that she has aspiration pneumonia secondary to her weakness, one can argue that she could recover from the acute episode, as this is a reversible cause. If, by contrast, it is solely her ALS that is affecting her so severely, one can argue for end-stage ALS, in which case she would probably not be a candidate for either intensive care or resuscitation. The ethical dilemma therefore rests on whether the patient would be able to survive a stay in intensive care without major permanent sequelae, and whether she could return to an ethically defensible level of functioning. If this is not thought possible, she would not be a candidate for intensive care, and this decision must be explained to the patient. Conversations about ceilings of treatment should always be discussed with the patient, although ultimately they rest on a medical judgement. Of course, this can create a difficult situation, because the patient is probably greatly affected by the situation and it is life-threatening. It must be explained, as far as possible, that the risks associated with an intensive care stay in her present condition and with her terminal progressive disease would not be beneficial for her. The relatives should also be contacted. |
| 230830 | I would assess whether the patient should be admitted to the intensive care unit depending on the following factors: her age, comorbidities, current performance status, how advanced the disease is so far, and her current critical condition. In view of this patient, I would not think she should be admitted to intensive care, though I would of course discuss this with the neurology senior on-call doctor. |
| 242740 | Overall, I would reflect on the patient’s own wishes. I would speak with the patient about the prognosis and the implications of a stay in intensive care, and about wishes concerning possible resuscitation/intubation. The most important things are the patient’s own wishes and the medical assessment in relation to performance status and expected remaining life. It is not certain that the patient would ever come off the ventilator again, and she should be informed thoroughly about this. On the other hand, she would most likely go into respiratory arrest because of hypercapnia if nothing is done. One could perhaps discuss trying less invasive respiratory aids such as NIV, but that is not sustainable in the longer term. The patient should be allowed to speak with her relatives about this before a decision is made—to say goodbye, etc. |
| 215654 | The ethical considerations involve taking account of the patient’s wishes, while at the same time making a medical judgement about whether she is a candidate for intensive treatment. From a medical point of view one has to weigh advantages and disadvantages. We know this is an incurable disease that will only get worse. I would therefore approach the situation by considering the wishes of the patient and relatives, while reflecting on the patient’s condition and on how far intensive treatment could be used to relieve symptoms. I would handle such a situation by discussing it with the senior on-call doctor and other colleagues, and by remembering to involve and explain the final medical decision to the relatives. |
| 221681 | The patient has ALS, which is a disease that does not improve. I would therefore not expect her condition to improve even with intensive treatment, and I do not think she is a ventilator candidate either. However, she is only 55 years old, so I also think it is difficult simply to say that she should now receive palliative care; I would therefore contact my senior on-call doctor and the palliative care team for help in managing the situation. I do not feel that I yet have enough experience myself to assess whether a patient in her condition is a candidate for intensive treatment, so I would use the support available to me. |
| 219912 | Hopefully a doctor has already spoken with the patient in a calm phase about the possibility of ventilator treatment and her thoughts about this. If the patient is still awake, lucid and able to discuss it, this should of course be attempted. But if not, I would start by seeing whether I can find an older doctor colleague to discuss it with. One must take account of disease progression and functional status—is the patient newly diagnosed with few symptoms, or is she far advanced in the disease course? The key question is whether the patient can tolerate an intensive care course (including muscle loss, pain, discomfort and anxiety) and still have a meaningful life afterwards. I would discuss the case with the intensivists, including possible palliative treatment for air hunger. How severe is the respiratory insufficiency? Are there other treatment options, such as NIV? Speak with the relatives, if possible. |
| 220535 | It is important to speak with the patient early in the course about wishes concerning intensive treatment and resuscitation. The patient has been diagnosed with a severe, fatal disease that will only progress. It is likewise important to involve the family early in conversations about the level of treatment. This is a difficult situation, because one wants to do everything possible to help the patient, but in some cases it may also be better to hold back and let the patient have peace. From a medical point of view, it is worth considering whether the patient would be able to tolerate possible intensive treatment. It is not certain that the patient has the capacity even to come off, for example, ventilator treatment. As a junior doctor, I would always discuss a difficult situation with older colleagues so that there are more eyes on the case and, hopefully, the best decision is made for the patient. |
| 236679 | (First consider reversible causes of respiratory insufficiency, such as infection or pulmonary oedema.) Ethical considerations: with ALS the respiratory problems will worsen continuously. This means the patient will probably need CPAP/ventilator treatment for the rest of her life. One must therefore consider the patient’s own wishes, palliative treatment, and how much time the relatives might still have with the patient. |
| 226556 | This is a difficult situation, because the patient can be placed on a ventilator, but there is no hope of improvement such that she could come off it again. Therefore, it is very important to speak with the patient and relatives about their wishes and to plan treatment in the end stage of the disease well before one is standing in this situation. |
| 233219 | I would consider the following: can she tolerate intensive treatment? What would her situation be after intensive treatment? Is there any likelihood at all that she would come off the ventilator again, and if so, in what condition would she return? Her respiratory muscles are already involved, which is prognostically poor. I would also investigate whether the patient herself has taken a view on intensive treatment and possible resuscitation. I think it would be important to involve the patient’s husband and the patient herself, if possible. In addition, I would call the senior on-call doctor, preferably the neurology senior on-call doctor, in order to gain a better overview of the probability that the patient would come through an intensive care course well. |
| 185723 | ALS is progressive and the prognosis is poor, and therefore intensive care or ventilator treatment would not benefit the patient in the longer term. Symptom-relieving treatment could be offered instead. |
| 225152 | There are several ethical considerations in relation to this condition, including the fact that the patient’s condition is not curable and will worsen within a short time. The patient will probably be placed on a ventilator and, with high probability, will not come off it again. The information provided does not describe how greatly the patient’s functional level is affected, though increased risk of infection, reduced swallowing reflex and reduced muscle strength are stated, and this may indicate impaired function. The situation could be approached, among other ways, by speaking with the relatives about the impact on the patient of being placed on a ventilator, the prognosis for ALS, and how much more should be done for the patient. |
| 230747 | We can keep the patient alive by ventilator treatment, but because of the progressive course of ALS the respiratory problems will not improve, so it is by no means certain that the patient can be taken off the ventilator. One must assess the patient’s own wishes about this, and perhaps the patient should instead be placed on comfort care with sedatives and analgesics so that she may quietly fall asleep. Naturally, this choice requires a conversation with the patient and relatives, as well as a conversation with an older colleague, who might also be present during the discussion. |
| 235133 | I would of course always contact my senior on-call doctor. A decision should have been made for all patients as to whether the patient is a candidate for intensive care and ventilation; this is a medical decision, but it is something that should be discussed with the patient before such situations arise. In this case it may be very difficult because the patient is relatively young, and I think that neither the patient herself nor the relatives wish to ‘stop treatment’ and move to comfort care. We know that the disease will only progress, so when one weighs the consequences against the benefits, it may not make much difference in the end whether she comes to intensive care or not. I would contact my senior on-call doctor and make a plan that I can communicate to the nurse and the relatives. I would call anaesthetics regarding the CO2 retention if I were alone and needed help. It would, of course, depend on the plan. |
| 244228 | The patient has a progressive condition with no curative treatment and no prospect of substantial improvement. If she is admitted to intensive care and placed on a ventilator, there is a high probability that she will never come off the ventilator again. It must therefore be carefully considered whether ventilator treatment should be offered; if so, home ventilation must also be considered. If the patient is somewhat less unwell, NIV could be considered, but just like invasive ventilation, this would represent an expected deterioration in a progressive disease, and she would then require home NIV. The relatives, and of course the patient herself if possible, should be spoken to about how far one should go with treatment and how they wish the patient’s final period to look: should one keep her alive and try to prolong life as much as possible, or when should treatment be stopped? |
| 239521 | The patient is not a candidate for ventilation because she would not be able to come off a ventilator again owing to weakened respiratory muscles. However, I do think she should be given a place in intensive care, because intensive care is much better at relieving symptoms in the final period, as staff there are used to patients who are very unwell. It might perhaps be possible to offer a little CPAP or NIV, but this should be discussed with a more experienced colleague and perhaps an anaesthetist in relation to what would make sense to subject her to. Alternatively, one should at least relieve the patient’s symptoms in her final period. |
| 243069 | I would hope that the patient’s GP or the doctor responsible for her treatment had already spoken with the patient about what she and her family want in the final stage of life, and whether she/the family had already taken a view on resuscitation and intensive treatment. On the basis of her disease, I would not assess her as a candidate for intensive treatment or for resuscitation, because her prognosis would be poor and there would be a risk that she would not come off the ventilator again. I would instead prioritise palliative symptom-relieving treatment. One must try to explain this rationale to the nursing staff and to the family. It is preferable that this conversation has been had with the family in advance, so that one does not have to have it in the patient’s last hours, when it will of course be especially difficult for the relatives, who may also be overwhelmed by strong emotions and grief. As a junior doctor, I would also find it relevant to discuss these difficult decisions with an older colleague so that I am not left alone with the decision. |
| 221226 | I would consider whether the patient is a candidate for resuscitation/ventilator treatment. Hopefully, this episode has been preceded by a conversation with the patient and relatives about the matter. It is important that both the patient and the relatives participate in such a decision, with the necessary information. Whether the patient is a candidate depends on the stage of the disease, her wishes, and medical judgement. In such a case I would first check whether resuscitation/ventilator treatment is registered in the electronic record. I would also contact my senior on-call doctor, tell them about the episode and get good advice on the next step. In addition, I would contact the patient’s relatives and inform them about the deterioration. |
| 227977 | The patient’s prognosis is already poor, and she probably will not have the resources to get through rehabilitation after an intensive care course. This requires that the patient still has a fair amount of muscle function and that several years of life are expected. But since the patient already has comorbidity in the form of daily alcohol use and perhaps therefore reduced cognitive reserve, liver dysfunction and cardiac complications, one must consider whether it makes sense. What does the patient want? What does the family want? As a junior doctor I would need a consultant’s assessment of the patient’s functional level and indication for intensive care and ventilation; the major question is whether the patient can manage an admission to intensive care and the subsequent rehabilitation. So I would definitely seek advice from my senior on-call doctor, speak properly with the relatives, and check the patient’s notes to see whether a decision about intensive care has already been made. |
| 242431 | In the first place, before this happened, one should have taken a decision about the level of treatment. The patient is still relatively young and may have many good years ahead of her, and therefore she ought to receive full treatment if that is what she wants. It is also important to point out that there are still treatment options and that new medicines are being developed, so there may also be a cure in the future. However, it can be difficult to strike a balance between giving too much hope and being realistic about disease progression. In the current situation one must quickly follow the ABCDE approach and see whether one can identify a cause for the patient’s condition. The patient may need to be intubated and admitted to intensive care until she has been treated and returned to normal respiration. |
| 224543 | Unfortunately, the situation is futile and the patient will soon die. I would make sure that the relatives prepare themselves for the patient’s impending death. She is not a ventilator candidate, because she would not come off it again. If they are not already receiving psychological support, I would recommend this. The relatives need to prepare themselves to say goodbye to her. |
| 229746 | This is a condition with a terminal prognosis, in which the state will only worsen going forward. In addition, the patient’s current condition must be considered in relation to whether she is a candidate for intensive care, ventilator treatment and possibly resuscitation. The decision should as far as possible be made in dialogue with the patient and her relatives and with the involvement of an experienced senior on-call doctor. It is important to establish whether they have already considered the situation and what the treatment level should be. |
| 235698 | Respiratory difficulties are unfortunately to be expected in ALS and can be deeply anxiety-provoking and unpleasant for the patient. Therefore, morphine should be considered. As a junior doctor I would not make such a difficult decision alone, and I would therefore always discuss it with relevant and more experienced colleagues, including the neurologists and the intensive care doctors/anaesthetists. They can help to make the best decision. In addition, I am of course aware that the patient must be mentally clear. The patient must be able to say yes or no herself, and be able to say that treatment should be stopped along the way if she wishes. It also depends on what the patient herself wants, perhaps in consultation with her spouse. Some would prefer not to be connected to machines when they know that their time is in any case drawing to a close. Others would like to make use of as much of their remaining life as possible. Therefore, one should speak with them and hear their thoughts and reflections after informing them about the possibilities and the consequences associated with them. |
| 222558 | This is a difficult situation, because ALS is a highly progressive disease with a fatal outcome. Most people die because of respiratory failure. Converting the patient to mechanical ventilation would be to prolong the unavoidable fatal outcome. With CO2 accumulation the patient will develop respiratory arrest, because CO2 drives respiration, and she will simply stop breathing. The ethical questions concern what constitutes a dignified death and what constitutes a good death. In addition, it is important to have a conversation about the level of treatment when a patient is diagnosed with ALS, both with the patient herself and with the relatives. |
| 229246 | She is only a 55-year-old woman, but ALS is a disease one dies from, and therefore it must be assessed, in relation to the patient’s current functional level, whether cognitive function, general functioning and quality of life are so compromised that it would be more brutal than gentle to offer a treatment level that includes intensive care and possible resuscitation. The views of both the patient and, importantly in this disease, the relatives should also be taken into account. Infections do worsen the disease, however, and ALS patients also get more infections than the healthy population, so perhaps the pneumonia should be treated fully before anything further is decided. |
| 217169 | I think it is probably not appropriate for a patient with such a poor prognosis to go through what would likely be a hard course in intensive care, which would probably take a heavy toll on the patient, and even if she recovered, she would live with rather poor quality of life for the rest of her short remaining time. So one has to weigh the various considerations, because normally our starting point is to cure and treat all patients. One could seek advice from an older colleague and of course ask the patient or the spouse what their thoughts are about intensive care and possible resuscitation, and about the poor prognosis of her disease. Here it is important to show empathy for their situation and to have the conversation in a polite and respectful way. |
| 238053 | One must know whether the patient, and perhaps the relatives, have taken a view on whether she wants ventilation or intensive treatment. If the patient wants this, and the treating doctor and senior on-call doctor find it defensible, one can consider it. However, it is a balancing act between her current functional level and what one can expect her to return to after intensive treatment on top of her underlying disease and remaining life span. The relatives should of course be informed of these considerations and of the grounds for the decision. |
| 220737 | My ethical reflections are that the patient, with a disease such as ALS, has a very poor prognosis, a short remaining life span and poor quality of life, and that she is now far into the course and probably requires respiratory support in the form of NIV or ventilation. It is a hard course both for the patient and for the relatives, and she will only become worse and worse; one may therefore consider whether the best thing for the patient and relatives might be to move the patient to comfort care. If intensive care is contacted and the patient is placed on a ventilator, there is a high risk that she will spend the rest of her life connected to it and will need to remain in hospital or have care staff present around the clock. I would not handle a situation like this alone, and would therefore contact my senior on-call doctor/neurology senior on-call doctor, who can help clarify matters and make a decision together with the patient and relatives. In an acute situation, however, I would always approach the patient using the ABCDE framework and treat the patient’s symptoms with a view to ABCDE optimisation. |
| 234392 | In a patient with ALS—a progressive disease without curative treatment—ethical considerations mean taking account of the wishes of the patient and the relatives regarding intensive care, ventilation and resuscitation. Intensive care in an ALS patient with progressive functional impairment is not without substantial risks, and there is a high probability that such a course will leave the patient permanently dependent on a ventilator and support, without the possibility of regaining function. Before the intensive care unit is contacted, oxygen can reasonably be given on the ward while one speaks with the patient and relatives. If the patient is unable to speak for herself, it may be useful to speak with the relatives, who can advocate for her best interests. It is important that the patient and relatives are thoroughly informed about the extent of intensive treatment and the expected outcome, namely permanent dependence on care. The family should have the opportunity to talk it through, but as a doctor it is my responsibility to ensure that the treatment level is consistent with the patient’s expected functional level and likelihood of regaining an acceptable level of function. |
| 233670 | I am wondering whether my patient is even a candidate for intensive treatment. We know that her symptoms have worsened over the last few months, and her condition with pneumonia makes me suspect reduced lung function, for example in relation to coughing. When I admit my patients, I always discuss the treatment level with them, and I hope I also did so in this case, because I think this is a borderline case. I am uncertain whether it would benefit her to be placed on a ventilator if we cannot reverse things enough for her respiratory function to improve. I hope I have my senior on-call doctor to discuss it with. If we have not talked about treatment level, and this is the first time she has become this unwell, I would probably let the doubt benefit her and treat her intensively. I do not know what her own wishes are either, but that is an ongoing conversation with a patient like this. |
| 229675 | As an ALS patient, one will often end up needing ventilator treatment at the end of the illness, because the respiratory muscles can no longer maintain their function. In this case, if there is an explanation for the respiratory distress in addition to her disease, for example pneumonia, ventilator treatment may be necessary until the infection has been brought under control. I would first check whether a decision about intensive treatment has already been made during this admission; perhaps the doctor responsible for treatment has already spoken with the patient about this possibility. I would then contact my senior on-call doctor and hear what she thinks. As such a junior house officer, I should not make this decision on my own. If the patient is not a candidate for intensive treatment, I would inform the family of this in a gentle manner and explain how such a course might unfold. |
| 222770 | One should consider the ethical dilemmas associated with full active treatment—ventilator treatment, intensive care and resuscitation in the event of cardiac arrest—for a patient with severe classical ALS. The patient has a progressive disease with a poor prognosis. There is no curative treatment. Ventilator treatment might help the patient for the present, but it would merely prolong the suffering, and the chances of coming off the ventilator are hopeless. One should consider whether the patient should instead be placed on palliative care, even if that means that she dies from CO2 accumulation. To assess the matter more closely, I would contact a more experienced medical colleague regarding the treatment level and possibly have the conversation together with that colleague, the patient and the relatives as soon as possible in a private room. In the end it is a medical decision, but if there are wishes in relation to the above, these will of course be taken into account. |
| 239165 | Hopefully there has been a conversation with the patient on admission about the treatment level in relation to being a ventilator candidate and attempts at resuscitation. The patient is a relatively young woman but with a very unpleasant disease that only goes one way. I would consider what sort of life we would be saving her back to. It would be relevant to have a conversation with both the family and the patient, and explain to them what the outlook is and that we cannot cure the disease. Before I went in to speak with the family and the patient, I would discuss it with an older colleague in order to handle this difficult conversation in the best possible way. |
| 219872 | As the patient is now far advanced in her ALS progression and has respiratory problems, one can consider whether ventilator treatment is an option or not. Ventilator treatment could prolong the patient’s life, but she would probably never come off it again, which is why one must reconsider whether the patient should be subjected to this. Instead, one can give oxygen and perhaps a little morphine to relieve the worst discomfort for the patient. Patients should not be kept alive simply because one can do so, if there is no prospect of improvement and treatment would merely drag things out and do more harm than good. As a junior doctor, I would contact my senior on-call doctor regarding whether treatment should be initiated at all, and if so, which treatment. The family should be informed about how events are likely to unfold and whether they or the patient have any wishes. |
| 238948 | Oh dear, I think this is another difficult situation to be in. The patient is becoming increasingly unwell from a respiratory point of view, and it therefore has to be assessed whether she is a candidate for either NIV or ventilator treatment. I would contact my senior on-call doctor for advice and help in making that decision, because it is not something I have encountered before. However, I can say that the consideration I am making is whether the patient would be able to tolerate going onto a ventilator—or, in other words, whether she would be able to come off it again. My immediate view is that she would not, given her disease. But again, I would want the advice of my senior on-call doctor. |
| 236687 | I would check whether a decision has been made in the electronic record about the level of treatment—resuscitation and whether the patient is a ventilator candidate. If no decision has been made, and if it is possible to do so with the patient, this should be done. The outlook regarding the complications that follow from her diagnosis of classical ALS is similar to the case described above, and things will only worsen over time. One naturally wants to help everyone regardless of their situation. In addition, she is a young woman. The advantages and disadvantages of possible ventilation therefore have to be weighed. I would contact the intensive care department and discuss with them what should be done. In addition, I would also contact the neurology senior on-call doctor and seek help from them, and anaesthetics depending on what we decide. I would also speak with my colleagues afterwards if there were a need for that. |
